# Trochanteric fracture pattern is associated with increased risk for nonunion independent of open or closed reduction technique

**DOI:** 10.1186/s12877-022-03694-0

**Published:** 2022-12-22

**Authors:** Till Berk, Sascha Halvachizadeh, David Paul Martin, Christian Hierholzer, Dominik Müller, Roman Pfeifer, Gerrolt Nico Jukema, Boyko Gueorguiev, Hans-Christoph Pape

**Affiliations:** 1 Department of Trauma, University Hospital Zurich, Raemistrasse 100, 8091 Zurich, Switzerland; 2 University of Zurich, Harald-Tscherne Laboratory for Orthopedic and Trauma Research, Sternwartstrasse 14, 8091 Zurich, Switzerland; 3 Department of Orthopedics and Rehabilitation, University of Wisconsin, 1685 Highland Ave, Madison, WI 53705 USA; 4 Cantonal Hospital Frauenfeld, Pfaffenholzstrasse 4, 8501 Frauenfeld, Switzerland; 5 AO Research Institute Davos, Clavadelerstrasse 8, 7270 Davos, Switzerland

**Keywords:** Traumatic proximal femur fractures, Polytrauma, Nonunion, Delayed union, Risk factors, Geriatric trauma

## Abstract

**Purpose:**

Soft tissue injury and soft tissue injury as risk factors for nonunion following trochanteric femur fractures (TFF) are marginally investigated. The aim of this study was to identify risk factors for impaired fracture healing in geriatric trauma patients with TFF following surgical treatment with a femoral nail.

**Methods:**

This retrospective cohort study included geriatric trauma patients (aged > 70 years) with TFF who were treated with femoral nailing. Fractures were classified according to AO/OTA. Nonunion was defined as lack of callus-formation after 6 months, material breakage, and requirement of revision surgery. Risk factors for nonunion included variables of clinical interest (injury pattern, demographics, comorbidities), as well as type of approach (open versus closed) and were assessed with uni- and multivariate regression analyses.

**Results:**

This study included 225 geriatric trauma patients. Nonunion was significantly more frequently following AO/OTA 31A3 fractures (*N =* 10, 23.3%) compared with AO/OTA type 31A2 (*N =* 6, 6.9%) or AO/OTA 31A1 (*N =* 3, 3.2%, *p <* 0.001). Type 31A3 fractures had an increased risk for nonunion compared with type 31A1 (OR 10.3 95%CI 2.2 to 48.9, *p =* 0.003). Open reduction was not associated with increased risk for nonunion (OR 0.9, 95%CI 0.1 to 6.1. *p =* 0.942) as was not the use of cerclage (OR 1.0, 95%CI 0.2 to 6.5, *p =* 0.995). Factors such as osteoporosis, polytrauma or diabetes were not associated with delayed union or nonunion.

**Conclusion:**

The fracture morphology of TFF is an independent risk factor for nonunion in geriatric patients. The reduction technique is not associated with increased risk for nonunion, despite increased soft tissue damage following open reduction.

## Introduction

The constant increase of life expectancy leads to rising incidence of fractures of the proximal femoral [1]. According to the “International Osteoporosis Foundation” approximately 1.6 million low-energy fractures of the hip occur per year worldwide and that figure may rise to six million by 2050 [2]. The primary goal in the surgical treatment of geriatric trauma patients is early mobilization [3, 4]. Modern methods of surgical treatment result in regular healing of most hip fractures; though, due to the increasing incidence of TFF, even a small percentage of cases with nonunion in this field, can add up to a significant number [5, 6]. The most commonly performed surgical interventions for PFF consider extra- and intramedullary implants [7, 8]. Previous studies have reported, that most treatment failures of PFF occur in unstable fracture patterns, with a mal-reduction of the posteromedial cortex or in reverse oblique fractures [9–13]. Other studies indicated additional factors, that can contribute to implant failure, such as unfavorable fracture patterns and poor bone quality [9, 14]. In long-bone fractures, risk factors for nonunion were reported to include fracture displacement, advanced patient age and open reduction and internal fixation (ORIF) [15–17]. Based on the experience in treating shaft fractures, handling of soft tissue and minimal invasive surgery are associated with improved outcome [18, 19]. The aim of the present study was to identify predictors for nonunion in geriatric trauma patients with TFF.

## Methods

### Ethical consideration.

This retrospective cohort study was approved by the local institutional review board (Basec No.:2020–00,703). A consensus of data collection was obtained from all patients during hospitalization.

### Study population

#### Inclusion criteria

Geriatric patients, (age > 70 years) with TFF requiring surgical treatment between 2013 and 2020 at one academic Level 1 trauma center were eligible to be included in this study. Further inclusion criteria were complete data sets for fracture pattern, demographic, and surgical treatment. A complete follow up at our institution of one year after surgery was further an inclusion criteria.

#### Exclusion criteria

Patients with oncologic diseases related to the proximal femur resulting in pathological fractures, fractures not classifiable with the AO-classification and patients with infections were excluded from the study. Patients with additional ipsilateral lower limb fractures, open injuries, infection, associated vascular and neurologic injuries, and pelvic injuries were also excluded.

### Definitions, outcome and follow up

TFF were classified according to the AO/OTA-Classification [17]. The primary outcome of this study was nonunion following surgical fixation of TFF. The clinical evaluation of fracture healing is a combination of both radiographic- and clinical findings [20], patients with unremarkable clinical and radiological findings at 6 – 12 weeks postoperatively, and similar results from the rest of the consultations, were considered cured/healed. The present study defined delayed union as failed callus formation on 3 out of 4 cortices after 3 to 6 months, in absence of secondary fracture or implant loosening. Nonunion was defined in cases with lack of callus-formation after 6 months, implant breakage or requirement of revision surgery [21]. The follow-up appointments were arranged after 6 weeks, 12 weeks, 6 months and 12 months. The assessment during each follow-up included physical examination, pain-assessment, and requirement of gait support. Radiographic imaging to assess the state of fracture healing was performed at every appointment. The primary diagnosis was based on conventional radiographic diagnostics. The x-ray image was analyzed by two independent experts: one radiologist and one senior trauma surgeon. In cases of uncertainty a CT scan was performed for confirmation of the diagnosis.

### PFF treatment protocol

According to our in-house protocol, PFF are surgically treated with the patient in a supine position with traction. In all patients, closed reduction is performed initially under radiological control. If the reduction is inadequate in either anterior–posterior or lateral view, the treating surgeon decides to perform open reduction. In both cases the entry point of the intramedullary implant is the tip of the greater trochanter. In cases where sufficient reduction cannot be obtained, cerclage wires may be used at the discretion of the treating surgeon.

The standard implant used in all included patients was a Gamma3®-nail-System, with previous models. According to the in-house standards, A1.-1–3 and A2.1–2 were treated with a short nail. A2.3 and A3.1–3 are treated with a long version of the nail. Since this implant does not have a standard cement augmentation feature, no augmentation was performed in the study cohort. Other implants are available in the clinic with which augmentation can be achieved, however, these patients were excluded for better comparability of the cohort.

The postoperative rehabilitation includes full weight bearing, daily physiotherapeutic training, and optimized medical treatment as part of the geriatric comanagement [22].

### Statistical analysis

Continuous variables are summarized as mean with standard deviation (± SD). Categorical variables are displayed as count and percentages. Two groups of continuous variables were compared using the student’s t-test. For groups of binary variables the chi-square test was used. ANOVA was used when comparing more than two groups. Risk factors for outcome variables were assessed with univariate regression analysis and presented with odds ratio (OR) and 95% confidence interval (CI). First, the analyses investigated the association of fracture morphology on nonunion in exploratory analyses. Second regression models were built to analyze the effect of fracture morphology on nonunion. In multivariate analyses the effect of fracture morphology were corrected for statistical and clinical relevant variables. Variables included in the multivariate regressions analyses were either statistical relevant as defined by a *p-value* of 0.1 during exploratory analyses or clinical relevant: These include patient demographic, open reduction representing a soft tissue injury, and comorbidities. A *p-value* below 0.05 was considered statistically significant. Statistical analysis was performed using R software package (R Core Team (2019), R Foundation for Statistical Computing, Vienna, Austria; (https://www.R-project.org).

## Results

**Fig. 1:**
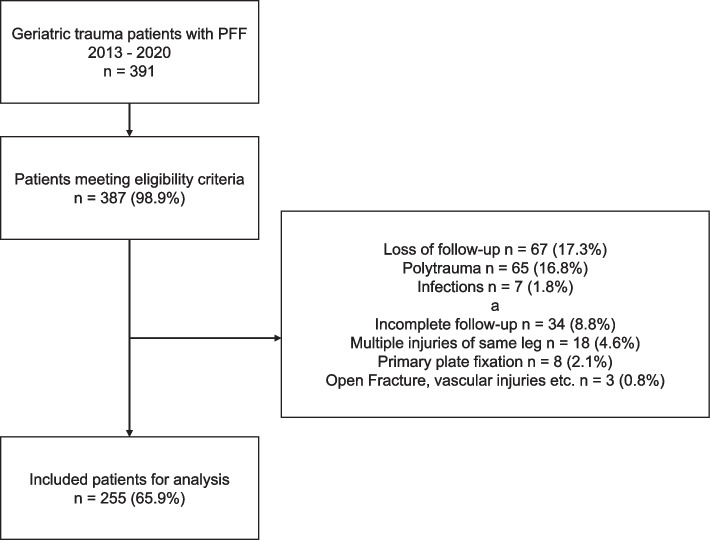
Flow chart of patient selection

Out of 387 eligible patients, 225 patients were included in this study (Fig. 1). Patient's age was 83.12 (SD 7.14) years with 160 (71.1%) of the patients being female. The majority of patients (*n =* 202, 89.2%) suffered a ground-level fall. The leading comorbidity was osteoporosis (*n =* 97, 43.1%). The overall length of stay (LOS) was 10.0 (SD 6.1) days.

The most common fracture type was AO/OTA 31A1 (*n =* 95, 42.2%), followed by AO/OTA 31A2 (*n =* 87, 38.7%) and 31A2 (*n =* 43, 19.1%). Out of the patients with AO/OTA 31A1 fractures, 19 (8.4%) suffered from type 1.1, 43 (19.1%) – from type 1.2, and 33 (14.7%) – from type 1.3 fractures. Type 2.1 fractures were identified in 31 (13.8%) patients, type 2.2 – in 33 (14.7%), and type 2.3 – in 23 (10.2%) patients. AO/OTA 31A3 fractures were distributed among 7 (3.1%) patients with type 3.1, 11 (4.9%) patients with type 3.2, and 25 (11.1%) patients with type 3.3 fractures.

Basic demographic data was comparable among patients' stratification according to the AO/OTA Classification. ORIF and ORIF with cerclage use was necessary in significantly more often in AO/OTA 31A3 fractures (ORIF: *n =* 27, 62.8%; ORIF with cerclage: *n =* 20, 46.5%) when compared to both AO/OTA 31A1 (ORIF: *n =* 5, 5.3%; ORIF with cerclage: *n =* 3, 3.2%) and AO/OTA 31A2 (ORIF: *n =* 20, 23.0%; ORIF with cerclage: *n =* 16, 18.4%), p ≤ 0.001 (Table 1). Patients with nonunion required more often ORIF (*n =* 8, 42.1%, *p =* 0.077) when compared with patients without nonunion. Factors such as osteoporosis, polytrauma or diabetes were not associated with nonunion (Table 2). LOS was significantly shorter in the remaining collective (9.63 ± 5.77 days) when compared to the patient collectives with nonunion (14.47 ± 8.32 days) and delayed union (12.55 ± 6.98 days, *p* ≤ 0.001).

**Table 1.**
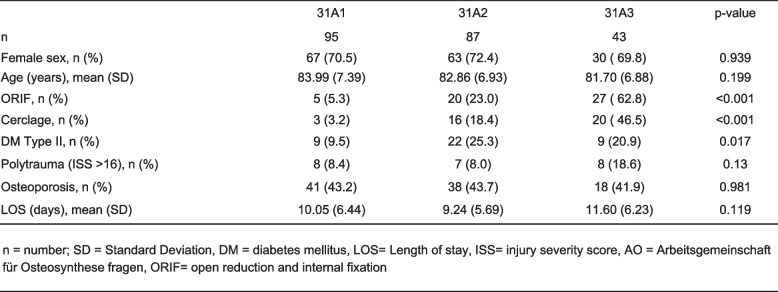
Demographic of study population stratified according to AOOTA classification of proximal femur fracture

### Delayed union

Delayed union was observed in 73 (32.4%) patients and occurred significantly often in patients suffering an AO/OTA 31A3 fracture (*n =* 31, 72.1%) compared with AO/OTA 31A2 (*n =* 26, 29.9%) or AO/OTA 31A1 (*n =* 16, 16.8%) fractures, *p <* 0.001 (Table 1).

Exploratory analysis revealed that patients with delayed union had significantly higher rate of ORIF (*n =* 27, 37%, *p =* 0.001) and required significantly more frequently cerclage (*n =* 19, 26.0%, *p =* 0.028) when compared with patients with regular healing time. After correction for patients demographics, comorbidity and open approach, AO/OTA type 31A2 had higher risk for delayed union (OR 2.2, 95%CI 1.1 to 4.7, *p =* 0.035) as did AO/OTA type 31A3 (OR 13.6, OR 4.9 to 37.2, *p <* 0.001) when compared with 31A1 fractures (Table 3). A univariate analysis of delayed union showed an odd ratio of 2.1 (95% CI 1.1 – 4.3, *p =* 0.039) for 31A2 fractures and 12.8 (95% CI 5.4–30.0) P ≤ 0.001) for 31A3 fractures when compared to 31A1 fractures.

**Table 2.**
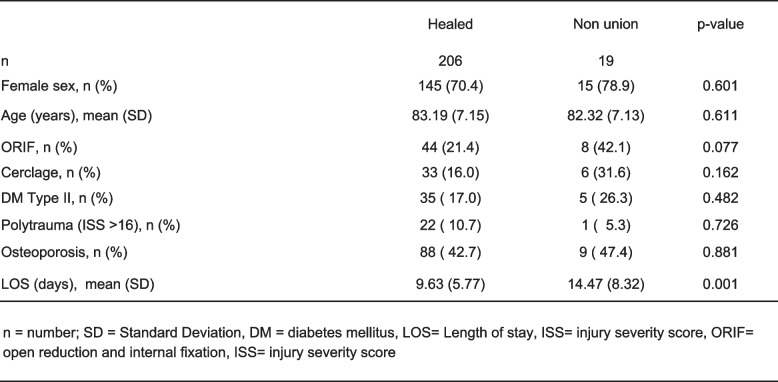
Risk factors for nonunion in exploratory analyses

**Table 3.**
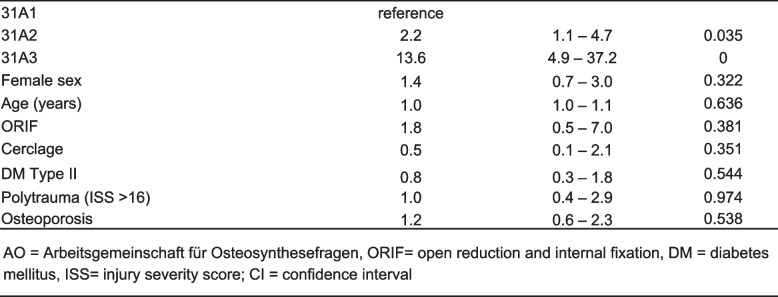
Prediction of delayed union in multivariate analyses

### Nonunion

Nonunion was observed with subsequent revision surgery – in 19 (8.4%) patients and was also indicated significantly more frequently following AO/OTA 31A3 fractures (*n =* 10, 23.3%) compared with AO/OTA type 31A2 (*n =* 6, 6.9%) or AO/OTA 31A1 (*n =* 3, 3.2%) fractures, *p <* 0.001 (Table 1). The AO/OTA type 31A3 fracture was an independent risk factor for nonunion when compared with AO/OTA type 31A1 fracture (OR10.3. 95%CI 2.2 to 48.9, *p =* 0.003) after correction for demographics, comorbidities, and type of approach. Open reduction was not associated with increased risk for nonunion (OR 0.9, 95%CI 0.1 to 6.1, *p =* 0.942) (Table 4).

**Table 4.**
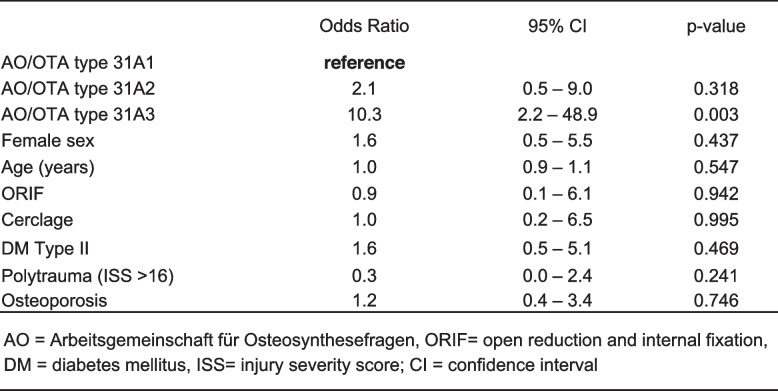
Prediction of non union in univariate and multivariate analyses

## Discussion

The increasing population of geriatric patients with PFF represents a special challenge in geriatric orthopedic trauma care. This study aimed to assess the risk factors for delayed- and nonunion of PFF in geriatric trauma patients and identified the following important aspects:

1. ORIF and cerclage applications were not associated with increased rates of nonunion in geriatric PFF
2. Severity of fracture morphology was associated with nonunion
3. AO/OTA 31 A3 fractures represent an independent risk factor for nonunion

The current definitions of delayed union and nonunion are inconclusive, since there is no agreement on the exact timing when the diagnosis should be made [23]. Various studies in the literature come to different timeframes regarding the diagnosis of nonunion. These vary between 6–8 weeks, 3 months and up to 6 months [24–26]. Average fracture healing varies from 6 weeks to 3 months [27]. The ability for weight-bearing with absence of pain/tenderness, as well as absence of pain/tenderness during examination/palpation are the most common clinical criteria for assessment of fracture healing, according to a review of fracture healing trials [28]. For the majority of patients, 3 months is a reasonable time to expect union [29]. A generally recognized definition of nonunion is a fracture that has not healed and will not heal without further intervention in the opinion of the attending physician [30]. Therefore, all patients diagnosed with nonunion were surgically revised in the current study.

Open reduction is associated with increased soft tissue damage and disruption of local vascularization that might lead to impaired healing due to disrupted local biology [31]. Further, the use of cerclage was reported as a risk factor for necrosis and disruption of vascularization [32]. The association between open reduction or use of cerclage with delayed union or nonunion is based on theoretical knowledge and has not been proven yet in a clinical setup [33]. In contrast to our study, a recently published meta-analysis reports favorable outcomes following use of cerclage, based on optimized reduction [34]. Therefore, the use of cerclage without increased risk of bone healing delay might be beneficial only for selected cases. Similarly, the effect of open reduction on PFF healing has not yet been conclusively proven in clinical setting [35].

Complex fracture patterns and the lack of medial cortical support, varus malreduction and residual displacement after reduction have been described as potential risk factors for nonunion of subtrochanteric femur fractures [36–38]. It appears that the fracture morphology represents an independent risk factor for healing delay. The focus should therefore remain on optimal surgical reduction, which can be achieved by ORIF when necessary, with support of cerclage wires if required.

## Strength & limitations

The retrospective design of the current work is related to certain well-known limitations. One might argue that this study has an increased risk for type 2 statistical error based on the low sample size. We believe that – based on the standardized treatment protocol and the comparability of the study groups – the presented results provide some evidence for the identification of risk factors for nonunion in geriatric patients with TFF. Further, one might argue that the definition of delayed union or nonunion might be discussed and that radiographic analyses might be biased. We therefore included clinical problems, such as pain or gait difficulties into the definition of delayed and nonunion. It might be possible that these modifications are too strict, however, we believe that these definitions are clinically more relevant. Finally, medication usage that might impair fracture healing were not taken into consideration in these analyses. These data were not available in our database. However, our exclusion criteria were very strict and most patients included suffered from most common comorbidities of geriatric patients (e.g. osteoporosis). We therefore assume that the number of patients who are under steroid therapy does not interfere with the present results.

## Conclusion

The initial reduction technique is independent of the nonunion rate in geriatric PFF fracture treatment. Increased fracture complexity represents an independent risk factor of delayed union. AO/OTA 31 A3 fractures are an independent risk factor for nonunion. The treating surgeon should therefore focus on optimal reduction and retention of the fracture and utilize well-known techniques to achieve this goal.

## Data Availability

The collected data will be stored securely in our institute for 10 years. During this period, they are still available on request. After the 10 years, the data will be deleted. However, all the datasets analyzed or generated during this study will be available from corresponding author on reasonable request.

## References

[1] Ozkan K, Eceviz E, Unay K, Tasyikan L, Akman B, Eren A (2011). Treatment of reverse oblique trochanteric femoral fractures with proximal femoral nail. Int Orthop.

[2] Socci A, Casemyr N, Leslie M, Baumgaertner M (2017). Implant options for the treatment of intertrochanteric fractures of the hip: rationale, evidence, and recommendations. Bone Joint J.

[3] Weller I, Wai E, Jaglal S, Kreder H (2005). The effect of hospital type and surgical delay on mortality after surgery for hip fracture. J Bone Joint Surg Br.

[4] Sener, Muhittin, et al. "Mortality and morbidity in elderly patients who underwent partial prosthesis replacement for proximal femoral fractures." Eklem hastaliklari ve cerrahisi= Joint diseases & related surgery. 2009:20(1):11–17. 19522686

[5] Kyle RF, Cabanela ME, Russell TA, Swiontkowski MF, Winquist RA, Zuckerman JD, Schmidt AH, Koval KJ Fractures of the proximal part of the femur. In (1994). Instructional Course Lectures, at the 61st Annual Meeting of the American-Academy-of-Orthopaedic-Surgeons.

[6] Kyle RF, Gustilo RB, Premer RF (1979). Analysis Of 622 intertrochanteric hip-fractures - retrospective and prospective-study. J Bone Joint Surg Am.

[7] Domingo L, Cecilia D, Herrera A, Resines C (2001). Trochanteric fractures treated with a proximal femoral nail. Int Orthop.

[8] Leung K, So W, Shen W, Hui P (1992). Gamma nails and dynamic hip screws for peritrochanteric fractures. A randomised prospective study in elderly patients. J Bone Joint surg Br.

[9] Haidukewych GT, Israel TA, Berry DJ (2001). Reverse obliquity fractures of the intertrochanteric region of the femur. J Bone Joint Surg Am.

[10] Sarathy MP, Madhavan P, Ravichandran KM (1995). Nonunion of intertrochanteric fractures of the femur - treatment by modified medial displacement and valgus osteotomy. J Bone Joint Surg Br.

[11] Wu CC, Shih CH, Chen WJ, Tai CL (1998). Treatment of cutout of a lag screw of a dynamic hip screw in an intertrochanteric fracture. Arch Orthop Trauma Surg.

[12] Bartonicek J, Skala-Rosenbaum J, Dousa P (2003). Valgus intertrochanteric osteotomy for malunion and nonunion of trochanteric fractures. J Orthop Trauma.

[13] Haidukewych GJ, Berry DJ (2003) Salvage of failed internal fixation of intertrochanteric hip fractures. Clinical Orthopaedics and Related Research:184–188. DOI 10.1097/01.blo.0000071753.41516.27 10.1097/01.blo.0000071753.41516.27 12838070

[14] Baumgaertner MR, Solberg BD (1997). Awareness of tip-apex distance reduces failure of fixation of trochanteric fractures of the hip. J Bone Joint Surg Br.

[15] Parker MJ (1994). Prediction of fracture union after internal fixation of intracapsular femoral neck fractures. Injury.

[16] Esmailiejah, Ali Akbar, et al. "Treatment of humeral shaft fractures: minimally invasive plate osteosynthesis versus open reduction and internal fixation." Trauma Mon. 2015;20(3). 10.5812/traumamon.26271v2 PMC4630601 26543844

[17] Müller, M. E., Nazarian, S., Koch, P., & Schatzker, J. (2012). The comprehensive classification of fractures of long bones. Springer Science & Business Media.

[18] Fong K, Truong V, Foote CJ, Petrisor B, Williams D, Ristevski B, Sprague S, Bhandari M (2013). Predictors of nonunion and reoperation in patients with fractures of the tibia: an observational study. BMC Musculoskelet Disord.

[19] Vallier HA, Cureton BA, Patterson BM (2011). Randomized, prospective comparison of plate versus intramedullary nail fixation for distal tibia shaft fractures. J Orthop Trauma.

[20] Hak DJ, Fitzpatrick D, Bishop JA, Marsh JL, Tilp S, Schnettler R, Simpson H, Alt V (2014). Delayed union and nonunions: Epidemiology, clinical issues, and financial aspects. Injury.

[21] Willems A, van der Jagt OP, Meuffels DE (2019). Extracorporeal shock wave treatment for delayed union and nonunion fractures: a systematic review. J Orthop Trauma.

[22] Halvachizadeh S, Gröbli L, Berk T, Jensen KO, Hierholzer C, Bischoff-Ferrari HA, Pfeifer R, Pape H-C (2021). The effect of geriatric comanagement (GC) in geriatric trauma patients treated in a level 1 trauma setting: A comparison of data before and after the implementation of a certified geriatric trauma center. PLoS One.

[23] Bhandari M, Guyatt GH, Swiontkowski MF, Tornetta Iii P, Sprague S, Schemitsch EH (2002). A lack of consensus in the assessment of fracture healing among orthopaedic surgeons. J Orthop Trauma.

[24] Nayak NK, Schickendantz MS., Regan WD, Hawkins RJ. Operative treatment of nonunion of surgical neck fractures of the humerus. Clin Orthop Relat Res. 1995;313:200–5. 7641481

[25] Cadet ER, Yin B, Schulz B, Ahmad CS, Rosenwasser MP (2013). Proximal humerus and humeral shaft nonunions. J Am Acad Orthop Surg.

[26] McQueen MM (2008). Nonunions of the proximal humerus: their prevalence and functional outcome. J Trauma Acute Care Surg.

[27] Martin C, Guillen M, Lopez G (2006). Treatment of 2-and 3-part fractures of the proximal humerus using external fixation: a retrospective evaluation of 62 patients. Acta Orthop.

[28] Corrales LA, Morshed S, Bhandari M, Miclau T (2008). Variability in the assessment of fracture-healing in orthopaedic trauma studies. J= Bone Joint Surg Am.

[29] Angelini M, McKee MD, Waddell JP, Haidukewych G, Schemitsch EH (2009). Salvage of Failed Hip Fracture Fixation. J Orthop Trauma.

[30] Brinker MR, O'Connor DP, Monla YT, Earthman TP (2007). Metabolic and endocrine abnormalities in patients with nonunions. J Orthop Trauma.

[31] Teuben MPJ, Halvachizadeh S, Kalbas Y, Qiao Z., Cesarovic N, Weisskopf M, TREAT Research Group. Cellular activation status in femoral shaft fracture hematoma following different reaming techniques–A large animal model. Journal of J Orthop Res®. 2022. 10.1002/jor.25309 PMC9790649 35301740

[32] Perren SM (2002). Evolution of the internal fixation of long bone fractures: the scientific basis of biological internal fixation: choosing a new balance between stability and biology. J Bone Joint Surg Br.

[33] Kilinc BE, Oc Y, Kara A, Erturer RE (2018). The effect of the cerclage wire in the treatment of subtrochanteric femur fracture with the long proximal femoral nail: a review of 52 cases. Int J Surg.

[34] Kim C-H, Yoon Y-C, Kang KT (2022) The effect of cerclage wiring with intramedullary nail surgery in proximal femoral fracture: a systematic review and meta-analysis. European Journal of Trauma and Emergency Surgery:1–14 10.1007/s00068-022-02003-z 35618854

[35] Ghayoumi P, Kandemir U, Morshed S (2015). Evidence based update: open versus closed reduction. Injury.

[36] Beingessner DM, Scolaro JA, Orec RJ, Nork SE, Barei DP (2013). Open reduction and intramedullary stabilisation of subtrochanteric femur fractures: A retrospective study of 56 cases. Injury.

[37] Park SH, Kong GM, Ha BH, Park JH, Kim KH (2016). Nonunion of subtrochanteric fractures: Comminution or Malreduction. Pak J Med Sci.

[38] Shukla S, Johnston P, Ahmad MA, Wynn-Jones H, Patel AD, Walton NP (2007). Outcome of traumatic subtrochanteric femoral fractures fixed using cephalo-medullary nails. Injury.

